# Expression of choline and acetylcholine transporters in synovial tissue and cartilage of patients with rheumatoid arthritis and osteoarthritis

**DOI:** 10.1007/s00441-014-2036-0

**Published:** 2014-11-25

**Authors:** Janet Beckmann, Jan Schubert, Hans-Georg Morhenn, Veronika Grau, Reinhard Schnettler, Katrin Susanne Lips

**Affiliations:** 1 Laboratory of Experimental Trauma Surgery, Justus-Liebig University, Schubert Strasse 81, 35392 Giessen, Germany; 2 Laboratory of Experimental Surgery, Justus-Liebig University, Giessen, German; 3 Department of Trauma Surgery Giessen, University Hospital of Giessen-Marburg, Giessen, German

**Keywords:** Rheumatoid arthritis, Non-neuronal cholinergic system, Acetylcholine, Choline transporter-like proteins, Organic cation transporter, Human

## Abstract

Increasing evidence is showing that the non-neuronal cholinergic system plays an important role in the pathology of rheumatoid arthritis (RA). Choline transport into the cell is the rate-limiting step for the synthesis of acetylcholine (ACh), which can be released directly or in vesicles from the cell. However, in the human joint little is known about choline import or the release of ACh from the cell. Thus, we analyze the expression of members of the organic cation transporter (OCT), of the newly discovered choline transporter-like (CTL) family and of classical neuronal components such as the high-affinity choline transporter (CHT1) and the vesicular ACh transporter (VAChT) in the synovium and cartilage of the human hip joint from patients with osteoarthritis (OA) and RA. OCT1, OCT3 and OCTN1 and all members of the CTL family were expressed in synovial and cartilage samples. The expression of CTL1 and CTL2 was localized in synovial macrophages and fibroblasts. CHT1 mRNA expression was detectable only in the synovium, whereas VAChT was completely absent in all samples. Therefore, in the human joint, choline transport into the cell and the release of ACh seems to be mediated mainly by members of the OCT and CTL family. Expression of transporters appears not to be influenced by the pathological state, as no differences have been detected between joints from OA or RA patients. Importantly, however, all necessary components for choline import and the release of non-neuronal ACh are present in the human joint.

**Electronic supplementary material:**

The online version of this article (doi:10.1007/s00441-014-2036-0) contains supplementary material, which is available to authorized users.

## Introduction

Acetylcholine (ACh) is commonly known as a neurotransmitter. Nevertheless, increasing research has recently concentrated on the importance of ACh as a signaling molecule in non-neuronal cells in which it can play a role in regulating various biological functions (Beckmann and Lips 2013). ACh exerts its functions by acting on muscarinic and nicotinic receptors present on effector cells. The expression of these receptors is, however, not an indication for the existence of a non-neuronal cholinergic system (NNCS) which is characterized by the ability of the cell to produce and release ACh. The synthesis of ACh from choline and acetyl coenzyme A is facilitated by the enzyme choline acetyltransferase (ChAT; Wessler et al. 2003). In some non-neuronal cells, ACh can alternatively be produced by carnitin acetyltransferase (CarAT; Lips et al. 2007; Tucek 1982). The uptake of choline into the cell is one of the essential and rate-limiting factors for the synthesis of ACh. Choline is a positively charged quaternary amine that requires carrier-mediated transport into the cell. The high-affinity choline transporter (CHT1) is the transporter with the highest affinity for choline and is Na^+^- and Cl^−^-dependent. CHT1 is required for choline uptake in neuronal cells and without CHT1, neurons are unable to synthesize ACh (Ferguson et al. 2004). Even though some non-neuronal cells have been shown to express CHT1 (Lips et al. 2003; Pfeil et al. 2003), many non-neuronal cells that synthesize ACh do not express this transporter, indicating that other mechanisms of choline transport must exist. Notably, members of Na^+^-independent polyspecific organic cation transporters (OCT1-3) of the solute carrier protein (SLC) 22 family have been shown to have low affinity for choline (Koepsell 2004). Choline can be taken up by OCT1 and by OCT2. OCT3, however seems not to be able to recognize choline as a substrate (Busch et al. 1996; Sweet et al. 2001). Recently, the family of choline transporter-like (CTL) proteins has been identified as Na^+^-independent transporters with intermediate affinity for choline (O'Regan et al. 2000). To date, five members of this transporter family, CTL1-5, have been identified (Traiffort et al. 2005) but have not been extensively studied as yet. The best-characterized member CTL1 shows ubiquitous expression (Michel and Bakovic 2012) and CTL1-dependent choline transport has been described in many different cell types, including non-neuronal (Uchida et al. 2009; Yabuki et al. 2009) and neuronal (Machova et al. 2009; Yamada et al. 2011) cells. Interestingly, CTL1 expression seems to be regulated by extracellular choline availability, as choline deficiency can lead to the down-regulation of this transporter (Michel and Bakovic 2009). CTL2 has been shown to be expressed in several tissues such as muscle, kidney, heart, lung and the inner ear (Traiffort et al. 2013). Its role in choline transport has recently been described for heterologously expressed human CTL2 in *Xenopus laevis* oocytes (Kommareddi et al. 2010) and in lung adenocarcinoma cells (Nakamura et al. 2010). Expression of CTL3 has been found in kidney, ileum, and colon, while CTL4 is predominantly present in intestine, stomach, and kidney (Traiffort et al. 2005). Little is known about expression of CTL5, which has been found to low extend in the brain and in the spinal cord (Traiffort et al. 2013) and in small cell lung carcinoma cells, where it has been shown to be involved in choline transport (Song et al. 2013). Most interestingly, in cancer cells CTL4 does not only facilitate choline uptake but further seems to be specifically linked to ACh synthesis and secretion, as knock down of CTL4 resulted in a significant reduction of ACh (Song et al. 2013). The efficient release of ACh is another important factor characterizing a functional NNCS. In neuronal cells, the vesicular ACh transporter (VAChT) is required for ACh secretion. VAChT mediates the storage of ACh vesicles from which ACh is quantally released (Erickson et al. 1994). VAChT expression and vesicular storage and release have only been reported in some non-neuronal cells such as pancreatic α-cells (Rodriguez-Diaz et al. 2011), endothelial cells (Kirkpatrick et al. 2001) and cardiomyocytes (Rana et al. 2010). In most non-neuronal cholinergic cells, ACh is not stored in vesicles but is directly released via transporters. Of the family of organic cation transporters, OCT1 and OCT2 have been revealed to be able to translocate ACh out of the cell in the human airway (Lips et al. 2005), whereas in the placenta, ACh release is mediated by OCT1 and OCT3 (Wessler et al. 2001). Recently, a new family of OCTs, the organic cation transporters novel (OCTN), has been identified in higher organisms (Eraly et al. 2004) and the family member OCTN1 has been demonstrated to catalyze the transport of ACh (Pochini et al. 2012). Further, the mediatophore, a protein of 220 kDa consisting of 15-kDa proteolipid subunits of the vacuolar H^+^-ATPase, is thought to be involved in ACh exocytosis (Fujii et al. 2012; Israel and Dunant 1998).

Evidence is increasing that the cholinergic system can play an important role in the pathology of rheumatoid arthritis (RA; Pan et al. 2010). Depending on mode, time-point and immune status, the administration of nicotine has been shown to ameliorate experimental arthritis (Lindblad et al. 2009; van Maanen et al. 2009; Yu et al. 2011). However, the role of the α7 nicotinic receptor, which is known to function in the anti-inflammatory cholinergic pathway (Tracey 2009), is still being discussed controversially in this regard (van Maanen et al. 2010; Westman et al. 2010).

In general, little is known about the NNCS in the human joint. Grimsholm et al. in 2008 were able to show the expression of ChAT and the α7 nicotinic receptor in synovial tissue of the human knee joint in patients with RA and osteoarthritis (OA). A study of our own group confirmed the expression of α7 nicotinic receptor, other subunits of nicotinic receptors and various isotypes of muscarinic receptors (Schubert et al. 2012) even though we could not clearly determine the mRNA expression of ChAT, the ACh-synthsizing enzyme CarAT was clearly detectable. With regard to choline and ACh transporters, we could further identify the expression of OCT1 and OCT3 in the synovial tissue of the knee joints of RA and OA patients.

In the present study, we analyze the expression of various choline and ACh transporters, with special regard to the newly discovered choline transporter-like proteins, in the human joint. Even less information is available on the expression of the NNCS in cartilage. Thus, in addition to the synovial tissue, we also analyze samples of cartilage for choline and ACh transporter expression.

## Materials and methods

### Patients and samples

Synovial tissue and cartilage was obtained from RA and OA patients at the time of hip joint replacement. All patients fulfilled the criteria of the American College of Rheumatology Classification for RA (Arnett et al. 1988) and OA (Altman et al. 1986). Written informed consent was obtained from all participants. The study was performed according to the Declaration of Helsinki and was approved by the local ethical committee.

### RNA isolation and qualitative reverse transcription plus the polymerase chain reaction (RT-PCR)

Total RNA was extracted from the samples by using the RNA Lipid Tissue Mini Kit (Qiagen, Hilden, Germany). Removal of genomic DNA contamination and subsequent reverse transcription was performed with the Quantitect Kit (Qiagen) according to the manufacture’s protocol. Reactions without addition of reverse transcriptase were performed as controls (reverse transcription negative, RT-). AmpliTaq Gold polymerase (Applied Biosystems, Darmstadt, Germany) was used for cDNA amplification with gene-specific primers (Table 1; MWG, Ebersberg, Germany). For electrophoretic separation, RT-PCR products were subjected to 2 % TRIS-acetate-EDTA agarose gel electrophoresis.

**Table 1:** Human primer pairs used for qualitative an real-time RT-PCR (*CHT* high-affinity choline transporter, *CTL* choline transporter-like, *OCTN* organic cation transporters novel, *OCT* organic cation transporter, *VAChT* vesicular acetylcholine transporter, *βMG* β2-microglobulin)

Gene	Primer sequence	Length (bp)	Annealing temperature (°C)	Accession number
CHT1	ATCCCAGCCATACTCATT	168	59	NM_021815.2
	CAGAAACTGCACCAAGACCA			
CTL1	GTTCACTTGGAGGCACAGGT	206	62	BC049203.1
	GGCGATGGTAAGAGCAACAC			
CTL2	AAACCCTTGGCCCGGAGATGCT	160	62	BC040556.1
	GCCGCGCCTCTAGGACTCCAT			
CTL3	TCCTTGGCCTGTGTATCCTCGCA	130	62	AL540829
	ACACCGCAGACAAACAACAATCCCA			
CTL4	TGCCTACTGGGCCATGACTGCT	138	62	BC014659
	ACAAGGTGGGCCGTGGGGTT			
CTL5	CTGCTGAAGGAAGGAAGCAAAGCCA	138	62	BC051740
	AGGTACCCCCGATGTCGCCAA			
OCTN1	CTGTGGAGGAGCTAAATCCCCTGAA	149	59	BC028313
	AGGAGCATCCAGAGACAGAGCAA			
OCT1	GACGCCGAGAACCTTGGG	198	55	NM_003057
	GGGTAGGCAAGTATGAGG			
OCT2	TCGTCCATCGTCACCGAGT	302	59	NM_003058
	TATCTCCGCCCAACAAATC			
OCT3	GGAGTTTCGCTCTGTTCAGG	216	55	NM_021977
	GGAATGTGGACTGCCAAGTT			
VAChT	TACCCTACGGAGAGCGAAGA	157	59	U10554
	CTGTAGAGGCGAACATGACG			
βMG	TCTCTCTTTCTGGCCTTGGAG	134	59	NM_004048
	CAACTTCAATGTCGGATGGA			

### Real-time RT-PCR

Real-time RT-PCR analysis was performed by using gene-specific primers (Table 1, MWG), SYBR Green PCR Mastermix (Qiagen) and the I-Cycler IQ^TM5^ detection system (Bio-Rad, Munich, Germany). Standard and melt curves were performed to determine PCR efficiency and specificity of amplification, respectively. Mean cycle thresholds (Ct) values were normalized to the reference gene β2-microglobulin (βMG). Neuroblastoma cell line SH-SY5Y or colon carcinoma cell line CaCo-2 were used as positive controls.

### Immunohistochemistry

Synovial tissue and cartilage samples were fixed in 4 % phosphate-buffered paraformaldehyde (Merck, Darmstadt, Germany). Before being embedded in paraffin, cartilage samples were demineralized in 0.281 M TRIS-buffer containing 10 % ethylene diamine tetra acetic acid (Merck). Sections of 3 μm in thickness were cut and deparaffinized in a decreasing series of alcohols. For heat-induced antigen retrieval, sections were subjected to pressure-cooking in citric buffer (pH 6.0) for 15 min. After the blocking of endogenous peroxidase with H_2_O_2,_, sections were incubated with 1 % bovine serum albumin (BSA; Sigma Aldrich, Taufkirchen, Germany) and 10 % human serum (Sigma Aldrich) in phosphate-buffered saline (PBS) for 30 min at room temperature.

### Single-stain immunohistochemistry

Slides were incubated with the indicated primary antibody (Table 2) in antibody diluent (Dako, Hamburg, Germany) and 10 % human serum (Sigma Aldrich) overnight at 4 °C. Sections were subjected to biotinylated rabbit anti-mouse (1:150) or biotinylated goat anti-rabbit (1:800) secondary antibody (both from Dako) diluted in PBS containing 1 % BSA and 5 % human serum for 30 min at room temperature. After further treatment with Vectastain ABC Elite Kit (Vector Laboratories, Burlingame, Calif., USA) according to the manufacturer’s instructions, staining was visualized by using Nova Red (Vector Laboratories). Counterstaining was performed with hematoxylin and DePex (Serva, Heidelberg, Germany) was used for cover-slipping. Negative controls were performed without incubation of the primary antibodies and by using specific peptides (Table 2) for preabsorption overnight at 4 °C. To test the specificity of the antibodies further, Western blot analysis was performed (see Electronic Supplementary Material). Sections were evaluated with a photomicroscope (Axiophot-2, Zeiss, Jena, Germany) equipped with a digital camera (DC500, Leica, Bensheim, Germany).

**Table 2:** Antibodies and blocking peptides used for single-stain immunohistochemistry (*CHT* high-affinity choline transporter, *CTL* choline transporter-like, *OCT* organic cation transporter)

Primary antibody	Peptide
Mouse anti-human CTL1 (1:100); Abgent (#AT3924a)	CTL1 blocking peptide (200 μg/ml); Abgent (#BP14061c)
Mouse anti-human CTL2 (1:400); Acris (#AM21005PU-N)	CTL2 recombinant protein (200 μg/ml); Acris (#H00057153-Q01)
Rabbit anti-rat CHT1 (1:10,000; Lips et al. 2002; 92 % homology to human)	Rat CHT1 peptide (200 μg/ml; Lips et al. 2002)
Rabbit anti-human OCT1 (1:150); Aviva Systems Biology (#ARP41516_T100)	OCT1 blocking peptide (200 μg/ml); Aviva Systems Biology (#AAP41516)
Rabbit anti-rat OCT3 (1:300); Alpha Diagnostics (#OCT31-A; 94 % homology to human)	OCT3 blocking peptide (200 μg/ml); Alpha Diagnostics (#OCT31-P)

### Double-stain immunohistochemistry with two mouse monoclonal primary antibodies

Primary antibodies were diluted in PBS containing 1 % BSA and 10 % human serum and incubated with the sections overnight at 4 °C. For detection of the first primary antibody (Table 3), sections were incubated with rabbit anti-mouse IgG (1:25; Dako) followed by monoclonal mouse alkaline phosphatase/anti-alkaline phosphatase (APAAP; 1:25; Dako) for 30 min at room temperature each. Bound alkaline phosphatase was visualized by using Fast Blue chromogen (Sigma Aldrich). Antibodies were detached from the tissue by using another step of pressure-cooking. Sections were treated again for 30 min with 1 % BSA in PBS followed by overnight incubation at 4 °C with the indicated second primary antibody (Table 3). After incubation with peroxidase-labeled rabbit anti-mouse antibody (1:70; Dako), staining was visualized by using 3,3’ diaminobenzidine (DAB, Sigma Aldrich). Sections were cover-slipped with Kaiser’s glycerol gelatine (Merck, Darmstadt, Germany). For control of staining, both primary antibodies were omitted or each primary antibody was omitted separately.

**Table 3:** Antibodies used for double-stain immunohistochemistry with two mouse monoclonal primary antibodies (*CTL* choline transporter-like)

First primary antibody (blue)	Second primary antibody (brown)
Mouse anti-human CD68 (Clone PGM1; 1:200); DAKO	Mouse anti-human CTL1 (1:100)
Mouse anti-human CD163 (Clone EDHu-1; 1:200); AbD Serotec (#MCA1853T)	Mouse anti-human CTL1 (1:100)
Mouse anti-human prolyl-4-hydroxylase-β (Clone 3-2B12; 1:80); Acris (#AF0910-1)	Mouse anti-human CTL1 (1:100)
Mouse anti-human CTL2 (1:200)	Mouse anti-human CD68 (Clone PGM1; 1:300)
Mouse anti-human CTL2 (1:200)	Mouse anti-human CD163 (1:300)
Mouse anti-human CTL2 (1:200)	Mouse anti-human prolyl-4-hydroxylase-β (Clone 3-2B12; 1:80)

### Double-stain immunohistochemistry with mouse and rabbit primary antibodies

A mixture of mouse and rabbit primary antibodies (Table 4) was diluted in antibody diluent (Dako) and incubated with the sections overnight at 4 °C. After a 1-h treatment with alkaline-phosphatase-labeled swine anti-rabbit antibody (1:30; Dako), staining was visualized with Fast Blue chromogen. Subsequently, sections were incubated with biotinylated rabbit anti-mouse secondary antibody (1:150; Dako) and staining was visualized by using Vectastain ABC Elite Kit and DAB. Kaiser’s glycerol gelatine was used for cover-slipping. For control of staining, both antibodies were omitted or each primary antibody was omitted separately.

**Table 4:** Antibodies used for double-stain immunohistochemistry with mouse and rabbit primary antibodies (*CarAT* carnitin acetyltransferase, *CTL* choline transporter-like)

Primary rabbit antibody (blue)	Primary mouse antibody (brown)
Rabbit anti-human CarAT (1:100); LSBio (#LS-C167021/52635); preabsorption control with blocking peptide (LS-E8292/40 – LSBio)	Mouse anti-human CTL1 (1:100)
Rabbit anti-human CarAT (1:100); LSBio (#LS-C167021/52635); preabsorption control with blocking peptide (LS-E8292/40 – LSBio)	Mouse anti-human CTL2 (1:400)
Rabbit anti-human von Willebrand factor (1:10,000); Merck Millipore (#AB736)	Mouse anti-human CTL2 (1:400)

### Statistical analysis

SPSS software (SPSS Institute, Chicago, Ill., USA) was used for statistical analysis. Delta Ct values were compared by the global non-parametric rank-sum Kruskal-Wallis test. If significant (*P* ≤ 0.05), the Mann–Whitney test was used for further analysis of statistical relevance.

## Results

### Classical neuronal components: CHT1 and VAChT

**Fig. 1:**
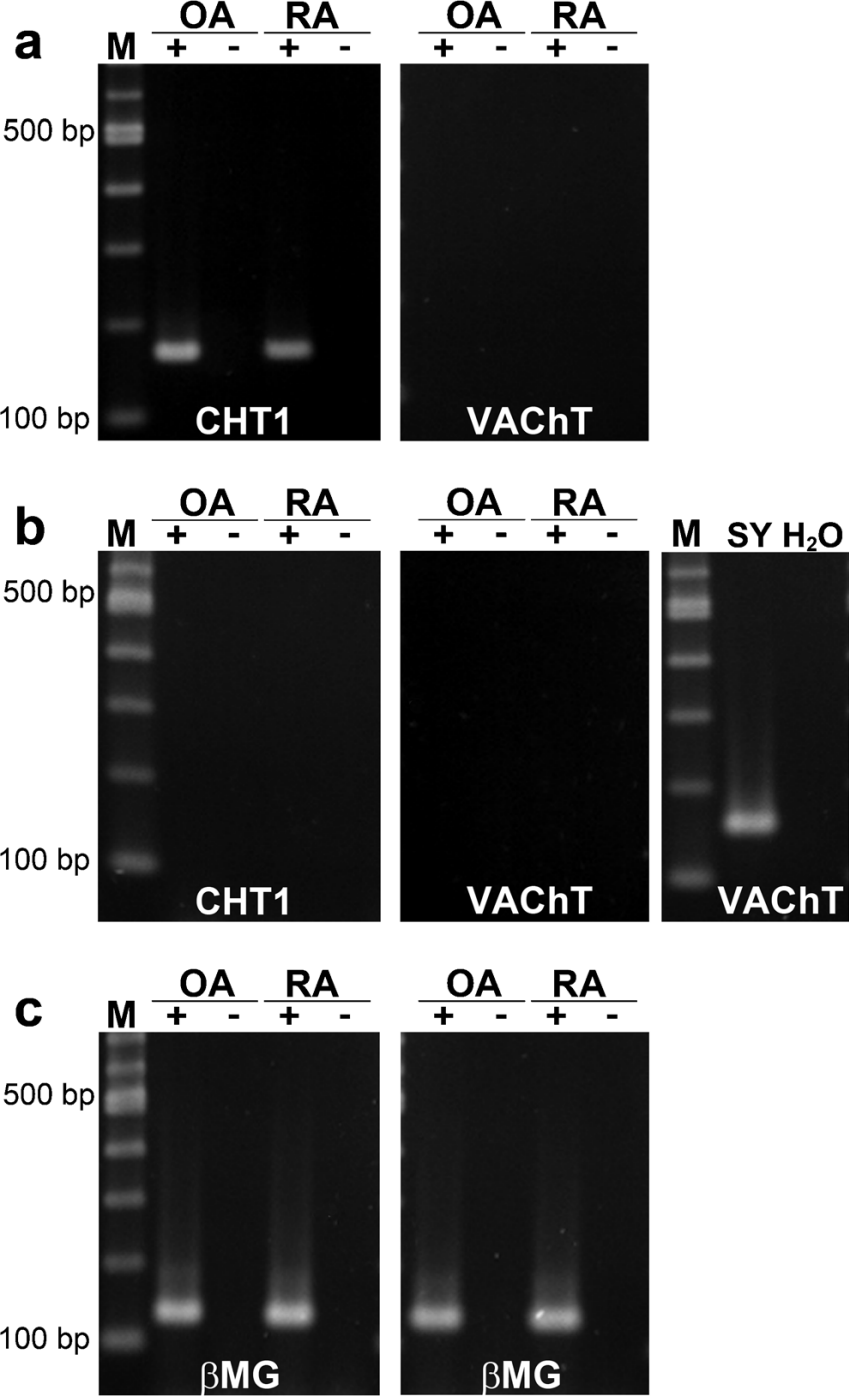
mRNA expression of the classical neuronal transporters *CHT1* (high-affinity choline transporter 1) and *VAChT* (vesicular acetylcholine transporter) in the human joint. **a**, **b** RT-PCR for *CHT1* and *VAChT* mRNA in synovial (**a**) and cartilage (**b**) samples from the hip of osteoarthritis (*OA*) and rheumatoid arthritis (*RA*) patients (*lanes M* 100 bp ladder, *lanes +* RT+ [reverse transcription with template], *lanes −* RT- [RT without template]). SH-SY5Y (*Sy*) was used as a positive control for *VAChT* expression and water (*H*_*2*_ *O*) was employed instead of cDNA as a negative control. **c** Representative RT-PCR for β2-microglobulin (*βMG*) in samples of synovia (*left*) and cartilage (*right*) from the hip of OA and RA patients

The classical neuronal choline transporter CHT1 was present in the synovial tissue of patients with OA and RA (Fig. 1a). In the joint cartilage, however, no *CHT1* mRNA expression was detectable in OA or RA patients (Fig. 1b). The mediator of ACh storage in vesicles, VAChT, was neither expressed in synovial tissue nor in cartilage samples of the human joint, irrespective of pathology (Fig. 1a, b). PCR was controlled by using neuroblastoma cell line SH-SY5Y as a VAChT-expressing positive control (Fig. 1b). For every used sample, PCR for βMG was performed to control RNA isolation and cDNA synthesis; an example is shown in Fig. 1c for synovial tissue and cartilage samples from patients with OA and RA.

### OCTs and OCTN1

**Fig. 2:**
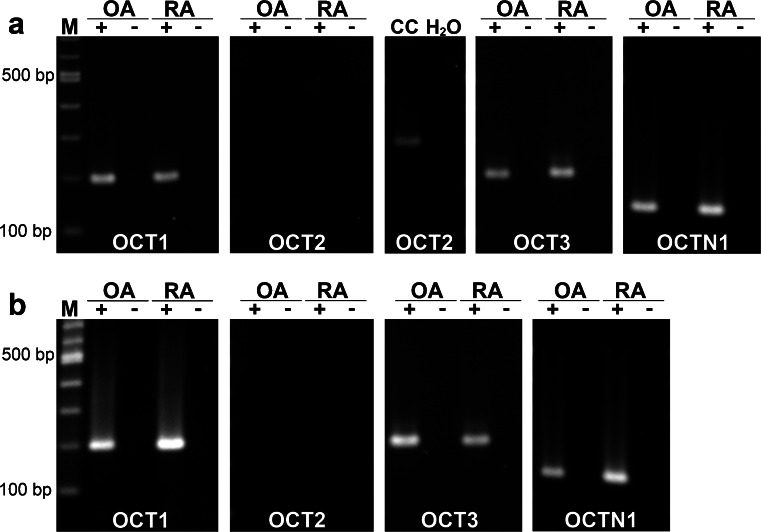
Expression of organic cation transporter mRNA in the human joint. **a**, **b** RT-PCR for organic cation transporter 1-3 (*OCT1-3*) and organic cation transporter novel (*OCTN1*) mRNA in synovia (**a**) and cartilage (**b**) from the hip of OA and RA patients (*lanes M* 100 bp ladder, *lanes +* RT+, *lanes −* RT-). CaCo-2 cells (*CC*) were used as a positive control for *OCT2* mRNA expression and water (*H*_*2*_ *O*) was employed instead of cDNA template as a negative control

In our previous study, we were able to show that *OCT1* and *OCT3* were expressed in the synovial tissue of the knee joint without showing a significant difference in expression levels between samples from RA and OA patients (Schubert et al. 2012). In the synovia of the hip joint, we could detect both transporters in samples from RA and from OA patients (Fig. 2a). *OCT1* and *OCT3* mRNA were also both present in all analyzed cartilage samples of the joints (Fig. 2b), irrespective of OA or RA pathology. Moreover, for the transporter OCTN1, mRNA expression was observed in synovial tissue (Fig. 2a) and cartilage (Fig. 2b) from all patients. *OCT2* mRNA, on the other hand, was detectable neither in synovial tissue (Fig. 2a) nor in cartilage (Fig. 2b) of any investigated sample.

### CTL proteins

**Fig. 3:**
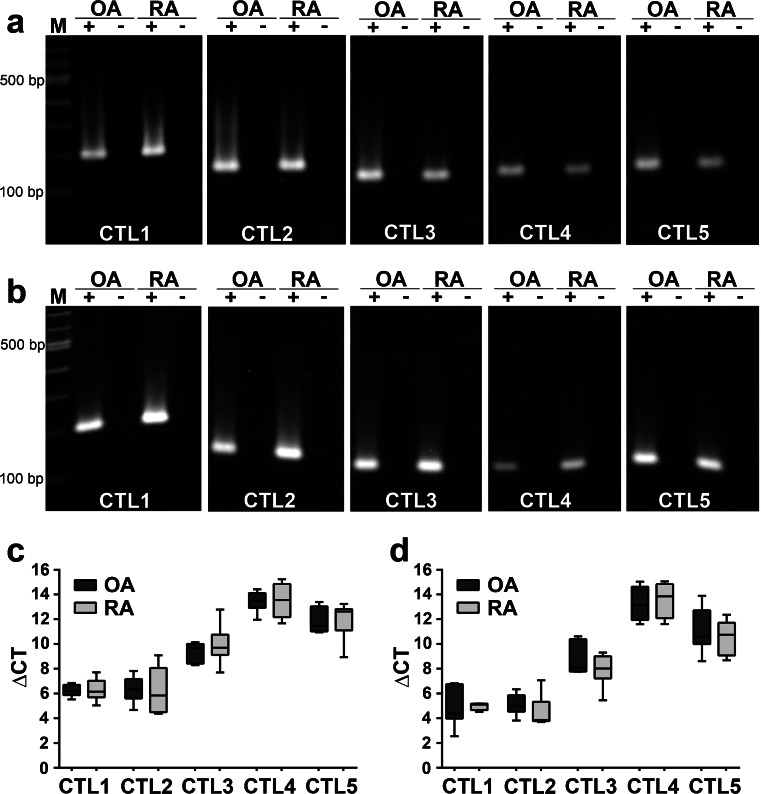
Expression of choline transporter-like (*CTL*) protein mRNA in the human joint. **a**, **b** RT-PCR for *CTL1-CTL5* mRNA in synovia (**a**) and cartilage (**b**) from the hip of OA and RA patients (*lanes M* 100 bp ladder, *lanes +* RT+, *lanes −* RT-). **c**, **d** Real-time RT-PCR for *CTL1-CTL5* mRNA in hip samples of synovia (**c**) and cartilage (**d**) of OA and RA patients (*n* = 5 each). Data were normalized to reference gene βMG and are presented as *box plots* with the median being indicated by a *solid line within the box*

All five family members of the CTL proteins were present in synovial tissue (Fig. 3a) and cartilage (Fig. 3b) of the human joint. No difference in mRNA expression levels of any CTL family member was observed between OA and RA patients by real-time RT-PCR (Fig. 3c-d). In all analyzed samples, *CTL1* and *CTL2* mRNA were the most prominently expressed, followed by *CTL3* and by a lower expression of *CTL4* and *CTL5* mRNA.

**Fig. 4:**
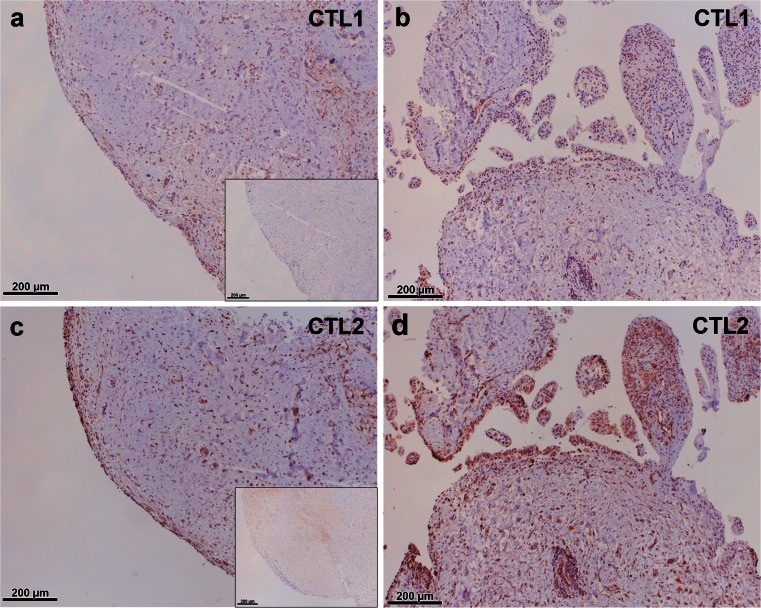
Immunohistochemical localization of CTL1 and CTL2 in the human joint. **a**, **b** Immunohistochemical staining for CTL1 in synovial tissue of the hip joint of OA (**a**) and RA (**b**) patients. *Insert* in **a** Preabsorption control with specific CTL1 peptide. **c**, **d** Immunohistochemical staining for CTL2 in synovial tissue of the hip joint of OA (**c**) and RA (**d**) patients. *Insert* in **c** Preabsorption control with specific CTL2 peptide

**Fig. 5:**
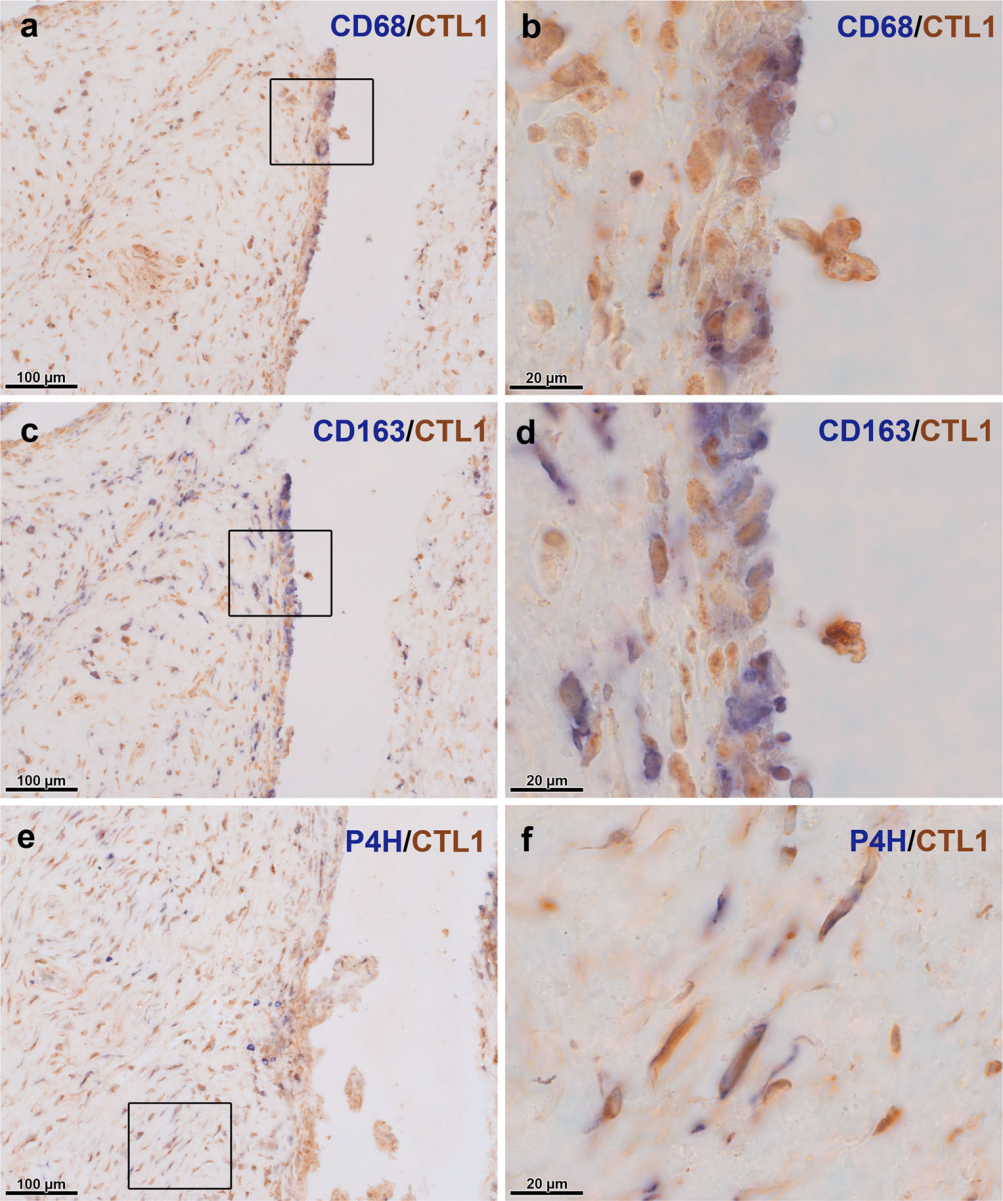
CTL1 is expressed in macrophage-like and fibroblast-like synoviocytes. **a**, **b** Double-staining for macrophage marker CD68 (*blue*) and CTL1 (*brown*) in synovial tissue. The *boxed area* in **a** indicates the region shown at higher magnification in **b**. **c**, **d** Double-staining for macrophage marker CD163 (*blue*) and CTL1 (*brown*) in synovial tissue. The *boxed area* in **c** indicates the region shown at higher magnification in **d**. **e**, **f** Double-staining for prolyl-4-hydroxylase-β (*P4H*, *blue*) and CTL1 (*brown*) in synovial tissue. The *boxed area* in **e** indicates the region shown at higher magnification in **f**

**Fig. 6:**
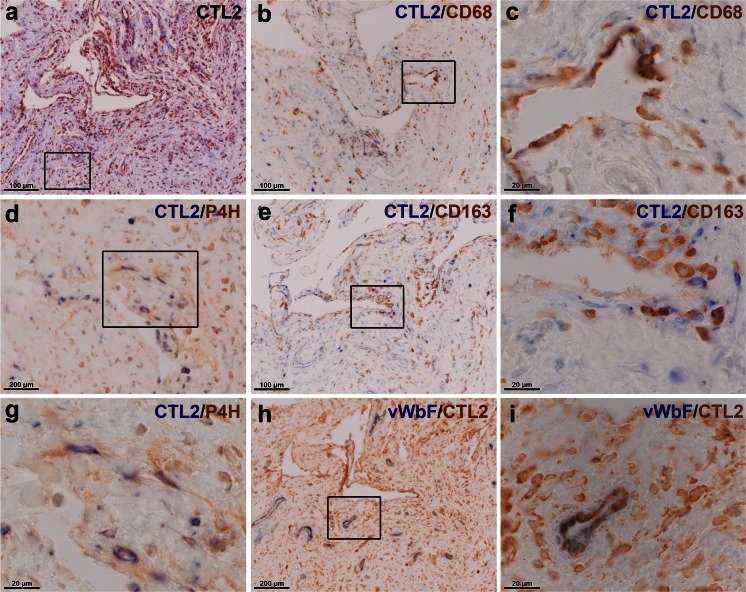
CTL2 is expressed by synovial macrophages and fibroblasts and by endothelial cells. **a** Single-staining for CTL2 in synovial tissue. The *boxed area* indicates the area of staining that is represented in **d**. **b**, **c** Double-staining for CTL2 (*blue*) and macrophage marker CD68 (*brown*) in synovial tissue. The *boxed area* in **b** indicates the region shown at higher magnification in **c**. **e**, **f** Double-staining for CTL2 (*blue*) and macrophage marker CD163 (*brown*) in synovial tissue. The *boxed area* in **e** indicates the region shown at higher magnification in **f**. **d**, **g** Double-staining for CTL2 (*blue*) and fibroblast marker, prolyl-4-hydroxylase-β (*P4H*, *brown*), in synovial tissue. The *boxed area* in **d** indicates the region shown at higher magnification in **g**. **h**, **i** Double-staining for CTL2 and endothelial cell marker, von Willebrand factor (*vWbF*). The *boxed area* in **h** indicates the region shown at higher magnification in **i**

**Fig. 7:**
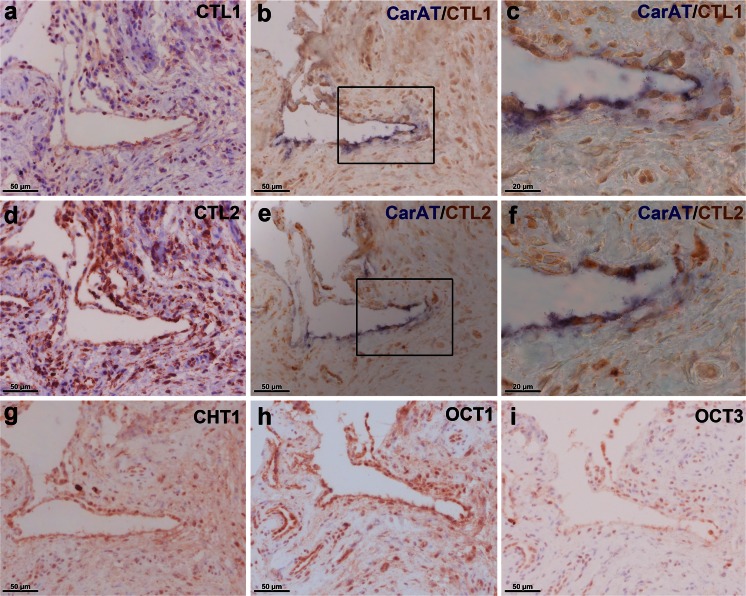
CTL1- and CTL2-positive cells express the ACh-synthesizing enzyme, carnitin acetyltransferase (*CarAT*). **a** Single-staining for CTL1 in synovial tissue. **b**, **c** Double-staining for CarAT (*blue*) and CTL1 (*brown*) in synovial tissue. The *boxed area* in **b** indicates the region shown at higher magnification in **c**. **d** Single-staining for CTL2 in synovial tissue. **e**, **f** Double-staining for CarAT (*blue*) and CTL2 (*brown*). The *boxed area* in **e** indicates the region shown at higher magnification in **f**. **g-i** Single-staining for CHT1 (**g**), OCT1 (**h**) and OCT3 (**i**) on synovial tissue

Little is known about the expression of CTL proteins in the joint. Thus, we were further interested in the protein localization of these choline transporters and performed immunohistochemical staining for the two most prominently expressed members, namely CTL1 and CTL2, in synovial tissue (Fig. 4). CTL1 and CTL2 protein were shown to be present in the synovial tissue of OA (Fig. 4a, c) and RA (Fig. 4b, d) patients and staining was mainly observed in the synovial lining layer but also in the subintimal layer. Double-staining of CTL1 with macrophage markers CD68 and CD163 and with fibroblast marker prolyl-4-hydroxylase-β localized the expression of this transporter to macrophage-like (Fig. 5a-d) and fibroblast-like (Fig. 5e, f) cells. Furthermore, the CTL2 protein was detected in synovial macrophages (Fig. 6b, c, e, f) and fibroblasts (Fig. 6d, g). Expression of CTL2 was further determined in endothelial cells by using double-staining for the von Willebrand factor (Fig. 6h, i). Moreover, the ACh-synthesizing enzyme CarAT was also detectable in CTL1-positive (Fig. 7b, c) and CTL2-positive (Fig. 7e, f) cells and the staining pattern of these two CTL proteins (Fig. 7a, d) was similar to that observed for CHT1 (Fig. 7g), OCT1 (Fig. 7h) and OCT3 (Fig. 7i).

## Discussion

**Fig. 8:**
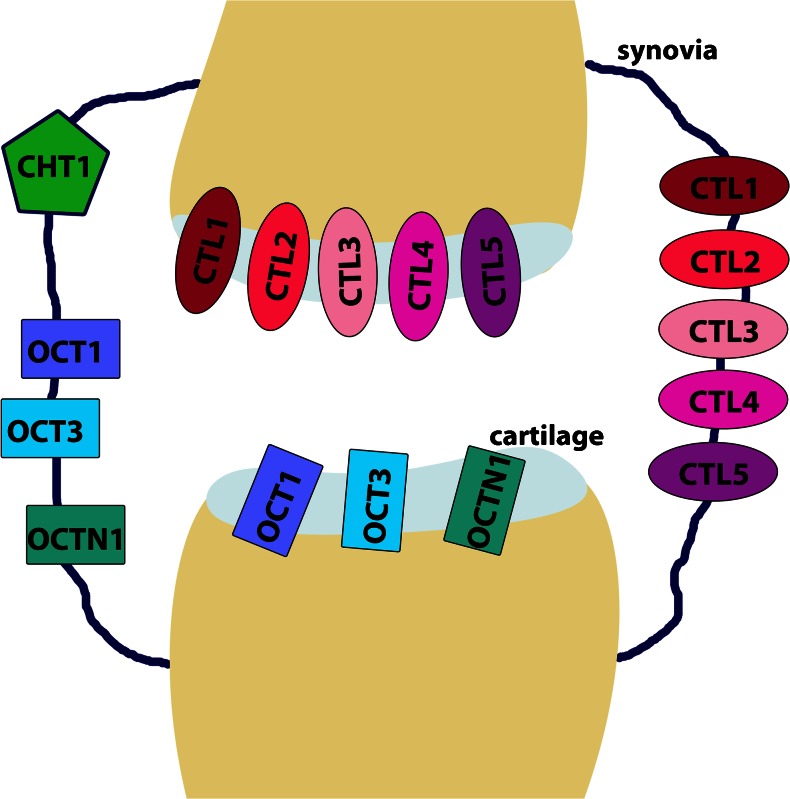
Representation of the expression of choline and acetylcholine transporters in the human joint. The transporters CTL1-5, OCT1, OCT3, and OCTN1 are expressed in the synovia and the cartilage of the human joint. The classic neuronal choline transporter CHT1 is only expressed in synovial tissue, and not in the cartilage of the joint

In the present study, we were able to show that all necessary components for the transport of choline into the cell and for the release of ACh are present in the synovial tissue and cartilage of the human hip joint (Fig. 8). At the mRNA expression level, no differences between samples of OA or RA origin are measurable, indicating that the pathological state might not influence the transport of choline or ACh in the human joint.

This is the first time that all members of the recently described family of CTL proteins have been shown to be expressed in the synovial tissue and cartilage of the human joint. CTL1, the best characterized member of the CTL family, is expressed the most prominently in both synovia and cartilage. This is in accordance with other tissues and cells such as keratinocytes in which CTL1 expression has also been found to be more pronounced than that of other CTL proteins (Uchida et al. 2009). We have been able to localize CTL1 protein expression to macrophage-like and fibroblast-like cells of the synovial tissue. As the specificity of CD68 as a macrophage marker has been critically discussed, we additionally chose to use CD163 to label macrophage-like synoviocytes in the tissue (Fonseca et al. 2002; Kunisch et al. 2004; Lau et al. 2004). Our finding is consistent with previous studies that identified CTL1 as being a constitutively expressed cell surface antigen on monocytic cells (Wille et al. 2001). Regulation of CTL1 in immune cells has only been reported for dendritic cells, as activation with calcium ionophores in these cells leads to the down-regulation of CTL1 (Wille et al. 2001). Further, human CTL1 has been shown to be up-regulated in several cancer cell lines (Wang et al. 2007) in myopathies and leukemias (Yuan et al. 2006). In the present study, however, no significant difference in CTL1 expression was observed between joints of OA and RA patients. For the human CTL1 gene, two splice variants have been characterized, encoding two proteins differing in a C-terminal peptide (Yuan et al. 2006). However, the specific roles and significance of the two CTL1 isoforms still have to be analyzed. Thus, in the present study, we used primers amplifying both transcript variants.

CTL2 was first discovered in the supporting cells of the inner ear (Zajic et al. 1991) and antibodies against CTL2 are reported to cause hair cell death and hearing loss (Nair et al. 2004). Notably, antibodies against CTL2 have been detected in 50 % of patients with autoimmune hearing loss (Kommareddi et al. 2009) indicating a prominent role of CTL2 in this and possibly other autoimmune diseases. Interestingly, a single nucleotide polymorphism in the CTL2 gene has been reported to encode for the human neutrophil alloantigen (HNA)-3a, which represents the target antigen for antibody-mediated transfusion-related acute lung injury (Curtis et al. 2010; Greinacher et al. 2010). In the human hip joint, we were able to localize CTL2 in macrophage-like and fibroblast-like cells and in endothelial cells of the synovial tissue. The latter finding is in accordance with previous studies in which CTL2 expression has been found in monocytes and endothelial cells (Flesch et al. 2013). No expression differences between joints of OA and RA patients have been observed. As for CTL1, alternative splicing results in two isoforms of the CTL2 protein differing in the first 12 amino acids of presumably the cytoplasmic N-terminus (Nair et al. 2004) and in one isoform that differs in its C-terminus and that might be epithelial-cell-specific (Kommareddi et al. 2010). To date, whether the various isoforms can exert diverse functions is unclear and thus, in the present study, we first took a general look at CTL2 expression by choosing primers amplifying all isoforms.

We were able to show that the other family members, namely CTL3, CTL4 and CTL5, are also expressed, though to a lesser extent, in synovial tissue and cartilage of the human joint. However, little is known about the functionality of these three transporters (Inazu 2014). Interestingly, CTL4 mRNA expression, which has been shown to be related to the synthesis and release of non-neuronal ACh in cancer cells (Song et al. 2013), is detectable in all analyzed samples, indicating a possible role of this transporter for ACh release also in the human joint.

From the family of polyspecific OCTs, both *OCT1* and *OCT3* mRNA are expressed in cartilage and in synovia of the human joint, whereas *OCT2* is not detectable in any analyzed sample. This observation is consistent with other studies reporting that OCT1 and OCT3 show a rather broad tissue expression pattern, whereas the distribution of OCT2 seems to be more restricted (Inazu 2014; Koepsell et al. 2007). The transporter OCTN1 has been shown to be expressed in a variety of tissues, especially immune-related tissue, such as spleen and bone marrow and in several immune cell types (Koepsell et al. 2007; Tokuhiro et al. 2003). Tokuhiro et al. (2003) reported that OCTN1 is expressed in synovial tissue of RA patients and in arthritic mice, whereas OCTN1 expression has not been observed non-arthritic control mice. Further, the induction of OCTN1 mRNA expression has been observed in human synovial fibroblasts isolated from RA patients after stimulation with tumor-necrosis factor-alpha (Tokuhiro et al. 2003). We were able to confirm the expression of OCTN1 in the synovial tissue of the hip joint of RA patients; however, in OA patients serving as the non-inflammatory control, the expression of OCTN1 is also detectable. The same can be observed for the analyzed cartilage samples in our study. Most interestingly, an association between functional variants of the OCTN1 gene and RA has been reported in the Japanese population leading to the hypothesis that the small nuclear polymorphism (SNP) in intron 1 of the OCTN1 gene lowers the affinity for the Runt-related transcription factor 1 (RUNX1), which regulates OCTN1 expression (Tokuhiro et al. 2003). However, the identification of OCTN1 as an RA susceptibility gene could not be verified in the Canadian (Newman et al. 2005), UK (Barton et al. 2005), or Spanish (Martinez et al. 2006; Orozco et al. 2006) population and could not be reproduced by another group working on the Japanese population (Kuwahara et al. 2005).

Taken together, these data suggest that the import of choline for ACh synthesis is facilitated mainly by OCT1 and members of the CTL family in the synovial tissue and cartilage of the human joint. In synovial tissue but not cartilage, the classic neuronal component CHT1 might also contribute to this process. The transport of ACh out of synovial or cartilage cells occurs directly and not via vesicles, as no VAChT expression is detectable. Members of the OCT family and CTL4 and OCTN1 might be responsible for mediating this direct release of ACh. In the synovial tissue, the transporters CTL1 and CTL2 have been localized to macrophage-like and fibroblast-like cells and other transporters show a similar expression pattern in this tissue. The presence of the ACh-synthesizing enzyme CarAT in cells expressing CTL1 and CTL2 provides a hint that these non-neuronal synovial cells produce ACh. Thus, all important components for the synthesis and release of ACh are present in the synovial tissue and cartilage of the human joint but no differences between OA and RA pathology are detectable.

## Electronic supplementary material

Below is the link to the electronic supplementary material.

ESM 1 (PDF 348 kb)

Fig. S1 (GIF 16 kb)

High resolution image (TIFF 1845 kb)
