# Supplementary material for: Expression of choline and acetylcholine transporters in synovial tissue and cartilage of patients with rheumatoid arthritis and osteoarthritis
Source: Cell Tissue Res. 2014 Nov 25;359(2):465–77. doi: 10.1007/s00441-014-2036-0 (PMC4320306; doi:10.1007/s00441-014-2036-0)
Supplement: Supplementary file 1 — (PDF 348 kb) [file 441_2014_2036_MOESM1_ESM.pdf]

Electronic supplemental material

Expression of choline and acetylcholine transporters in synovial tissue and cartilage of patients with rheumatoid arthritis and osteoarthritis

Janet Beckmann [a], Jan Schubert [a], Hans-Georg Morhenn [a], Veronika Grau [c], Reinhard Schnettler [a,b] and Katrin Susanne Lips [a]

[a] Laboratory of Experimental Trauma Surgery, Justus-Liebig University, Giessen, Germany

[b] Department of Trauma Surgery Giessen, University Hospital of Giessen-Marburg, Giessen, Germany

[c] Laboratory of Experimental Surgery, Justus-Liebig University, Giessen, Germany

Materials and methods

Wester blot analysis

For additional testing of antibody specificity, western blot analysis was performed. Synovial tissue samples were lysed in RIPA Buffer and the amount of total protein in the sample was determined using DC Protein Assay (BioRad). 50 µg of total protein were subjected to denaturing polyacrylamide gel electrophoresis and subsequent transfer to nitrocellulose membrane (GE Healthcare, Freiburg, Germany) using wet blot system. The membrane was blocked with Odyssey Blocking Buffer (LI-COR Biotechnology, Bad Homburg, Germany) for 1h at RT and incubated with mouse anti-human CTL1 (1:500), mouse anti-human CTL2 (1:500), rabbit anti-human OCT1 (1:1000) (for further specification od antibodies see Table 2) overnight at 4 °C. Mouse anti-human β-actin (1:1000; Sigma-Aldrich) was incubated for 1h at RT. For detection of primary antibodies fluorescent-labeled goat anti-mouse-800 (1:15000; LI-COR Biotechnology) and goat anti-rabbit-680 (1:15000; Biotium, Hayward CA, USA) were used and visualized using the Odyssey Sa Infrared Imaging System (LI-COR Biotechnology).

Fig. S1

Western blot analysis of synovial tissue samples to confirm antibody specificity. CTL1 (70 kDa), OCT1 (61 kDa) and CTL2 (37 kDa) could be detected in synovial tissue (S). β-actin (42 kDa) was used as control. M = prestained protein ladder (Themo Fisher, Schwerte, Germany)
