# Supplementary figures and images for: Expression of choline and acetylcholine transporters in synovial tissue and cartilage of patients with rheumatoid arthritis and osteoarthritis
Source: Cell Tissue Res. 2014 Nov 25;359(2):465–77. doi: 10.1007/s00441-014-2036-0 (PMC4320306; doi:10.1007/s00441-014-2036-0)

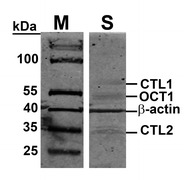

Supplement: Supplementary file 2 — (GIF 16 kb) [file 441_2014_2036_Fig9_ESM.gif]

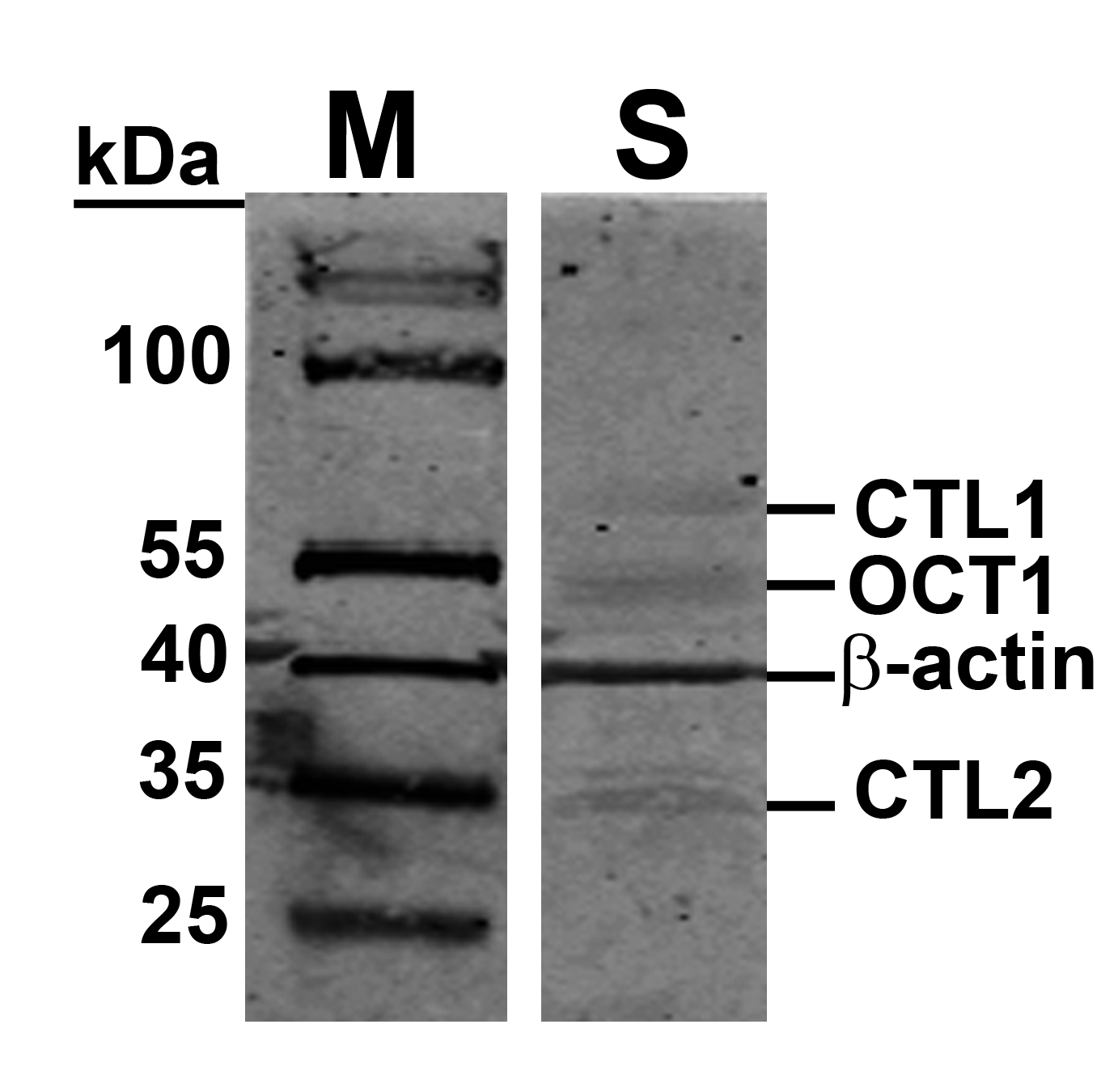

Supplement: Supplementary file 3 — High resolution image (TIFF 1845 kb) [file 441_2014_2036_MOESM2_ESM.tif]
